# Urinary monocyte chemoattractant protein 1 associated with calcium oxalate crystallization in patients with primary hyperoxaluria

**DOI:** 10.1186/s12882-020-01783-z

**Published:** 2020-04-15

**Authors:** Xiangling Wang, Gauri Bhutani, Lisa E. Vaughan, Felicity T. Enders, Zejfa Haskic, Dawn Milliner, John C. Lieske, On behalf of the investigators of the Rare Kidney Stone Consortium, Dean Assimos, Michelle Baum, Michael Somers, Lawrence Copelovitch, Prasad Devarajan, David Goldfarb, Elizabeth Harvey, Lisa Robinson, William Haley, Mini Michael, Craig Langman

**Affiliations:** 1 Division of Nephrology and Hypertension, Department of Internal Medicine, Mayo Clinic, 200 First Street SW, Rochester, MN 55905 USA; 2 Division of Biomedical Statistics and Informatics, Department of Health Sciences Research, Mayo Clinic, Rochester, MN USA; 3 Department of Laboratory Medicine and Pathology, Mayo Clinic, Rochester, MN USA

**Keywords:** Crystallization, Glomerular filtration rate, Monocyte-chemoattractant protein 1, Primary hyperoxaluria, Renal damage

## Abstract

**Background:**

Patients with primary hyperoxaluria (PH) often develop kidney stones and chronic kidney disease. Noninvasive urine markers reflective of active kidney injury could be useful to gauge the effectiveness of ongoing treatments.

**Methods:**

A panel of biomarkers that reflect different nephron sites and potential mechanisms of injury (clusterin, neutrophil gelatinase-associated lipocalin (NGAL), 8-isoprostane (8IP), monocyte-chemoattractant protein 1(MCP-1), liver-type fatty acid binding protein (L-FABP), heart-type fatty acid binding protein (H-FABP), and osteopontin (OPN)) were measured in 114 urine specimens from 30 PH patients over multiple visits. Generalized estimating equations were used to assess associations between biomarkers and 24 h urine excretions, calculated proximal tubular oxalate concentration (PTOx), and eGFR.

**Results:**

Mean (±SD) age at first visit was 19.5 ± 16.6 years with an estimated glomerular filtration rate (eGFR) of 68.4 ± 21.0 ml/min/1.73m^2^. After adjustment for age, sex, and eGFR, a higher urine MCP-1 concentration and MCP-1/creatinine ratio was positively associated with CaOx supersaturation (SS). Higher urine NGAL and NGAL/creatinine as well as OPN and OPN/creatinine were associated with higher eGFR. 8IP was negatively associated with PTOx and urinary Ox, but positively associated with CaOx SS.

**Conclusion:**

In PH patients greater urine MCP-1 and 8IP excretion might reflect ongoing collecting tubule crystallization, while greater NGAL and OPN excretion may reflect preservation of kidney mass and function. CaOx crystals, rather than oxalate ion may mediate oxidative stress in hyperoxaluric conditions. Further studies are warranted to determine whether urine MCP-1 excretion predicts long term outcome or is altered in response to treatment.

## Background

Primary hyperoxaluria (PH) is a group of autosomal recessive disorders characterized by recurrent urinary stones and nephrocalcinosis [1, 2]. Roughly half of PH patients present with advanced chronic kidney disease (CKD) at the time of diagnosis [1–3]. Three forms of PH have been recognized based on the underlying genetic defects. PH1 is caused by deficiency of hepatic peroxisomal alanine-glyoxylate aminotransferase (AGT) resulting from mutations in the *AGXT* gene, PH2 by deficiency of cytosolic and mitochondrial glyoxylate reductase/ hydroxypyruvate reductase, and PH3 by deficiency of mitochondrial 4-hydroxy-2-oxoglutarate aldolase [2].

The main biochemical defect in PH is overproduction of oxalate due to these enzyme deficiencies, primarily in the liver. The excess oxalate must then be excreted by the kidney [2]. In the kidney, oxalate can combine with calcium within renal tubules leading to nephrocalcinosis and kidney stones. Deposition of calcium oxalate within tubules and the interstitium and the immunological response can then produce progressive loss of renal function [4, 5]. Our recent study demonstrated that among PH patients higher urine oxalate excretion is an independent predictor of poorer renal outcome [6]. Thus, strategies to reduce urine oxalate excretion and/or calcium oxalate crystallization have been the cornerstone of PH treatment [2]. To date, improving fluid intake and use of neutral phosphate or potassium citrate as crystallization inhibitors have been the available options. Liver transplantation can replace the enzymatic defect in PH1 and perhaps also PH2, but carries inherent risk [7, 8]. Oral administration of oxalate degrading bacteria holds promise but remains an unproven strategy [9, 10]. Ribonucleic acid inhibition (RNAi)-based therapeutics that reduce hepatic oxalate production have been effective in animal models and are currently in clinical trials [4]. For any treatment plan, a sensitive marker of ongoing renal damage from oxalate and/or crystal would be a valuable tool to gauge the effectiveness of treatment in real time, and to assess whether oxalate excretion or crystallization has been sufficiently suppressed. Thus, in the current study we examined a panel of noninvasive candidate urine biomarkers of injury, which have been previously associated with inflammatory pathways, crystallization, and/or oxalate exposure in vitro or in vivo, in in order to determine if any correlated with urinary oxalate excretion, calcium oxalate supersaturation (CaOx SS), predicted proximal tubular oxalate concentration (PTOx), and estimated glomerular filtration rate (eGFR).

## Methods

### Study population

This study was approved by the institutional review board at the Mayo Clinic, Rochester Minnesota (IRB 13–0053) and was performed in accordance with the declaration of Helsinki and all patients were consented to participate. Our study population consisted of a cohort of 30 PH patients enrolled in the Rare Kidney Stone Consortium (RKSC) PH registry between 2004 and 2013 who had one or more biobanked urine specimens and no prior history of end stage renal disease (ESRD) or organ transplantation; among these, *n* = 24 were PH type 1, *n* = 4 were PH type 2, and n = 2 were PH type 3.

General information and clinical manifestations were abstracted from Registry data. As described previously [6], PH1 was confirmed by mutations of the *AGXT* gene, liver biopsy confirming deficiency of AGT, or by marked hyperoxaluria in combination with hyperglycolic aciduria in a patient with no identifiable secondary causes. PH2 was established by mutations of glyoxylate and hydroxypyruvate reductase gene (*GRHPR*), liver biopsy confirming deficiency of GRHPR enzyme, or hyperoxaluria in combination with hyperglyceric aciduria without identifiable secondary cause. PH3 was diagnosed by mutations of the 4-hydroxy-2-oxoglutarate aldolase gene (*HOGA1*) [6].

Laboratory results including serum creatinine, 24-h urine oxalate (UOx), calcium, citrate, creatinine, and CaOx supersaturation (CaOx SS) calculated by the computer program EQUIL2 [11], were extracted from registry data. Proximal tubular oxalate concentration (PTOx = (UOx* serum Cr* 4)/ UCr) was calculated as described by Worcester and colleagues [12]. Laboratory values were retained for analysis if they were collected within 3 months of the clinic visit corresponding to the biobanked urine specimen; however the vast majority of observations were collected on the same day as the clinical visit. eGFR was caculated using the Schwartz formula in children < 18 years old [13] and the CKD-EPI formula in adults [14].

A cohort of *n* = 47 non-stone forming adults in good general health without kidney disease or diabetes (22 women, 25 men, ages ranging from 23 to 77 years) who completed 24-h urine collections on a free choice diet served as the adult control group to establish an adult reference range for the urine biomarkers we studied. These samples were obtained from subjects participating in a reference value study. Although a lack of a prior history of kidney disease or kidney stones was confirmed, no other laboratory or dietary data was available for this group.

### Measurement of a panel of urine biomarkers

A 24 h urine sample was collected from patients at the time of an outpatient evaluation, centrifuged at 4 °C for 10 min at 2000 rpm, and the supernatant was frozen at -70 °C. For this study frozen aliquots were subjected to one prior freeze-thaw cycle. Urine monocyte chemoattractant protein 1 (MCP-1) was measured using the Quantikine Human chemokine (C-C motif) ligand 2/ monocyte chemoattractant protein-1 (CCL2/MCP1) ELISA kit (R&D Systems, Minneapolis, MN) as previously described [15]. Urine liver-type fatty acid binding protein (L-FABP) was measured using the L-FABP urinary ELISA kit (Agrutus Medical, Daiichi Fine Chemical Co., Ltd. Japan). Urine heart-type fatty acid binding protein (H-FABP) was measured using the H-FABP urinary ELISA kit (Hycult Biotech Inc., Plymouth Meeting, PA). Urine Neutrophil gelatinase-associated lipocalin (NGAL) was measured using the Bioporto immunoturbidometric assay run on a Roche COBAS autoanalyzer (Roche Diagnostics, Indianapolis, IN). Urine osteopontin (OPN) was measured using the OPN quantikine ELISA kit (R & D systems, Minneapolis, MN). Urine 8-isoprostane (8IP) was measured using the 8IP urinary ELISA kit (Cayman Chemicals, Ann Arbor, Michigan). Clusterin was measured using urinary clusterin ELISA kit (R & D, Minneapolis, MN). Urine creatinine was measured using the enzymatic creatinase assay on a COBAS auto analyzer (Roche Diagnostics, Indianapolis, IN). Duplicate measurements were performed for each ELISA measure, and those with a difference greater than 20% underwent repeat analysis. All urine biomarker concentrations were analyzed both with and without normalization to urine creatinine.

### Statistical analysis

All data are presented as the mean ± SD (range) or median (25 percentile, 75 percentile) for normally and non-normally distributed continuous variables, respectively and n (%) for categorical variables. Urine biomarker measures as well as UOx, urine calcium/Cr, urine citrate/Cr, and PTOx were natural log transformed for analysis to account for the skewedness of the distributions. Given the wide age range of this cohort which encompasses both pediatrics and adults, urine oxalate was adjusted for body surface area (BSA) and calcium and citrate were normalized to urinary creatinine. Univariate logistic regression models were used to compare urine biomarker levels between PH patients and controls at their first visit. Odds ratios (OR) and 95% confidence intervals (95% CI) were reported from these models. The associations between the urine biomarkers and 24 h urine oxalate excretion (UOx), calculated CaOx SS, urine calcium/Creatinine (CR), urine citrate/Cr, and calculated PTOx obtained from PH patients at one or multiple time points after diagnosis were evaluated using generalized estimating equations (GEE), which estimate robust standard error to take into account the correlated nature of the specimens within patients [16]. Parameter estimates from the GEE correspond to population averaged effects. GEE models were multivariate and in addition to each biomarker modeled separately, were also adjusted for the covariates age, sex and eGFR. The associations between the urine markers and eGFR were evaluated using multivariate GEE models adjusting for age and sex, PTOx, urine calcium/Cr, and urine citrate/Cr were only analyzed with biomarker concentrations, since urine creatinine had already been used to normalize these urine measures. *P*-values < 0.05 were considered statistically significant. All statistical analyses were performed using SAS, version 9.4 (SAS Institute Inc., Cary, NC).

## Results

A total of 114 frozen bio banked urine specimens collected over multiple visits from 30 PH patients were available between 2004 and 2013. Among these 30 patients, 22 had more than one urine specimen available, ranging from 2 to 9 specimens per person over the course of 0.5 to 8.4 years. Patients in this cohort had a mean ± SD age of 19.5 ± 16.6 years and mean ± SD eGFR of 68.4 ± 21.0 ml/min.1.73m^2^ at first visit, and consisted of 53% males (Table 1). Among these patients, *n* = 19 were < 18 at the time of their first visit, and 11 were > 18 years. The *n* = 47 adult controls that lacked a history of kidney disease or clinical stone events and were used for comparison to the adult PH patients ≥18 years old (*n* = 11) had a mean ± SD age of 48.2 ± 12.8 years and were 53.2% male. Compared to adult controls, adult PH patients had higher levels of NGAL and lower levels of Clusterin, MCP 1, OPN and H-FABP, both before and after adjustment for urinary creatinine concentration (*P* < 0.05 for all). Unadjusted 8 IP concentrations were slightly lower in PH patients compared to controls, (*P* < 0.01). L-FABP concentrations were lower in the PH group compared to controls (*P* = 0.004), while no significant difference was found in creatinine-corrected L-FABP and 8 IP between cases and controls (*P* = 0.66) **(**Table 2**).**

**Table 1:** Clinical and laboratory characteristics of 30 PH patients at first visit

Characteristics	
**Age, years**	19.5 ± 16.6 (3.4–68.2)
**Male**	16 (53.3%)
**PH Type**	
1	24 (80.0%)
2	4 (13.3%)
3	2 (6.7%)
**eGFR**^**a**^**, ml/min/1.73m**^**2**^	68.4 ± 21.0 (37.2–122.3)
**Urine Oxalate, mMol/24 h/1.73m**^**2**^	1.48 (1.05, 2.33)
**CaOx SS, DG**^**b**^	0.99 ± 1.18 (−1.81–2.57)
**PTOx, μmol/L**	51.1 (34.1, 78.6)
**Urine calcium/Cr ratio, mg/g**	5.98 (2.04, 8.68)
**Urine citrate/Cr ratio, mg/g**	29.0 (17.7, 55.0)

Data are presented as n (%) for categorical variables and mean ± SD (range) or median (25 percentile, 75 percentile) for continuous variables, depending on the skewedness of the distributions

^a^For patients> 18, the CKD-EPI equation was used to estimate GFR. For patients< 18, the Schwartz 2009 equation was used to estimate GFR

^b^DG: delta Gibbs, the units for CaOx SS

**Table 2:** Urine biomarker concentrations in adult PH cases (*n* = 11) at first visit and adult controls (*n* = 47)

Biomarker^**a**^	Cases, Median (IQR)	Controls, Median (IQR)	OR	(95% CI)	P
**Clusterin**	0.2 (0.1, 1.8)	8.6 (3.2, 16.1)	0.170	(0.048, 0.604)	**0.006**
**Clusterin/Cr**	0.5 (0.2, 3.2)	7.7 (3.9, 16.3)	0.247	(0.106, 0.574)	**0.001**
**NGAL**	16.0 (1.0, 23.0)	2.0 (2.0, 4.0)	2.897	(1.322, 6.346)	**0.008**
**NGAL/Cr**	34,483 (1818, 56,818)	2817 (1747, 3718)	4.611	(1.890, 11.254)	**< 0.001**
**8 IP**	589 (300, 793)	1172 (567, 1712)	0.188	(0.056, 0.637)	**0.007**
**8 IP/Cr**	1167 (1014, 1405)	1063 (695, 1302)	4.146	(0.615, 27.94)	0.14
**MCP 1**	38 (9, 81)	192 (96, 335)	0.159	(0.042, 0.596)	**0.006**
**MCP 1/Cr**	87 (38, 147)	165 (130, 204)	0.268	(0.075, 0.957)	**0.043**
**L-FABP**	2.0 (2.0, 2.0)	3.3 (2.9, 4.7)	~ 0	(~ 0, 0.003)	**0.004**
**L-FABP/Cr**	3.8 (2.9, 6.9)	3.6 (2.4, 4.8)	1.395	(0.358, 5.43)	0.63
**OPN**	43 (12, 141)	855 (386, 1618)	0.228	(0.093, 0.557)	**0.001**
**OPN/Cr**	93 (44, 284)	847 (432, 1441)	0.323	(0.160, 0.649)	**0.002**
**H-FABP**	293 (255, 1118)	2714 (1552, 4553)	0.149	(0.049, 0.450)	**< 0.001**
**H-FABP/Cr**	1063 (607, 2070)	2652 (1674, 3442)	0.201	(0.060, 0.666)	**0.009**

*P*-values in bold denote significance at the 0.05 level. IQR is Intraquartile range. OR is odds ratio. 95% CI is for 95% confidence interval

Models were fit using logistic regression

^a^Log transformations were taken for all urine biomarkers

Units of measure are Clusterin: ng/ml and μg/g Cr; *NGAL* ng/ml and ng/g Cr, *MCP1* pg/ml and pg/g Cr, *L-FABP* ng/ml and μg/g Cr, *OPN* ng/ml and μg/g Cr, *H-FABP* pg/ml and ng/g Cr

**Fig. 1:**
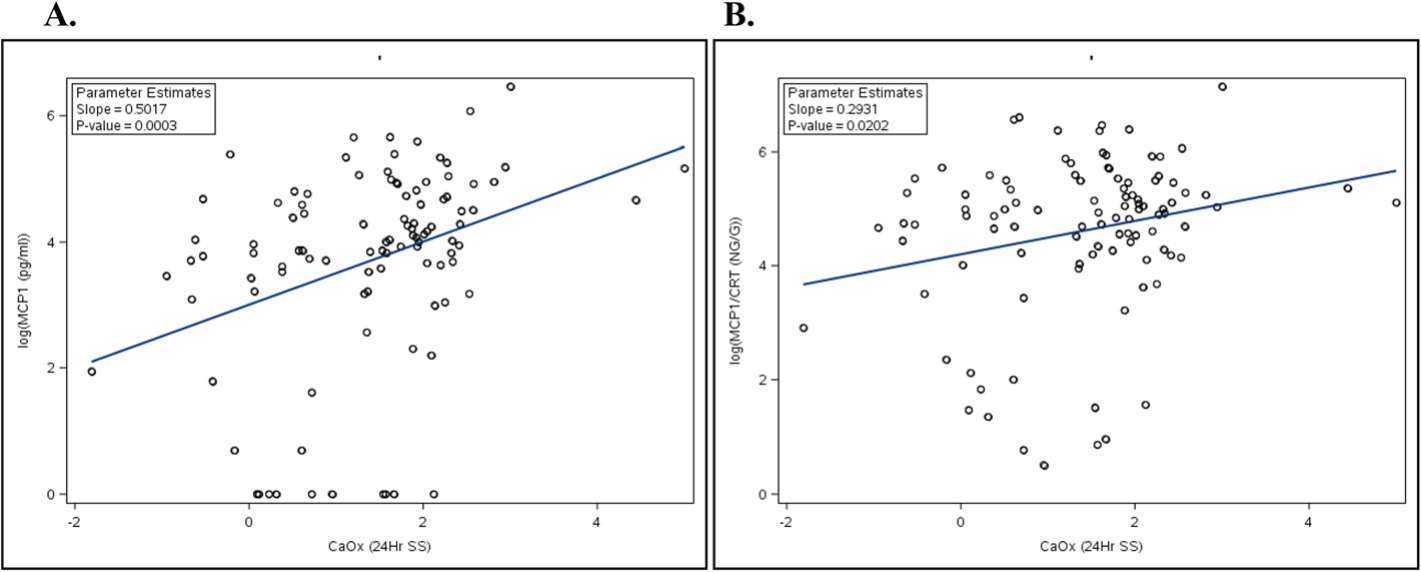
Association of CaOx SS with MCP-1. Log-transformed MCP-1 concentration (pg/ml) correlation with CaOx SS (Delta Gibbs units) (R^2^ = 0.12). Log-transformed MCP-1/Cr ratio (ng/ g creatinine) correlation with CaOx SS (Delta Gibbs units) (R^2^ = 0.05). In both panels data are shown for the PH cohort only

Among the 30 PH patients, both unadjusted and creatinine-adjusted MCP 1 levels were positively associated with CaOx SS (Fig. 1a and b). As shown in Table 3**,** this association remained even after adjustment for age, sex, and eGFR. 8 IP concentration was also positively associated with CaOx SS, while negatively associated with PTOx. The creatinine-corrected biomarkers L-FABP and H-FABP were both positively associated with PTOx; L-FABP was also negatively associated with CaOx SS while H-FABP was positively associated with urinary oxalate. As shown in Table 4, after adjustment for age and sex, both urinary NGAL and OPN were positively associated with eGFR, with and without adjustment for urinary creatinine.

**Table 3:** Association of urine markers with 24 urine oxalate excretion (Uox), CaOx supersaturation (CaOx SS), proximal tubular oxalate concentration (PTOx), urine calcium/Cr, and urine citrate/Cr, among n = 30 PH patients

Biomarker^**b**^	Uox^**b**^		CaOx SS		PTOx^**ab**^		Calcium/ Cr^**ab**^		Citrate/ Cr^**ab**^	
	Estimate (95% CI)	P	Estimate (95% CI)	P	Estimate (95% CI)	P	Estimate (95% CI)	P	Estimate (95% CI)	P
**MCP-1**	−0.195 (− 0.898, 0.509)	0.59	0.446 (0.207, 0.684)	**<.001**	− 0.135 (− 0.769, 0.498)	0.68	− 0.04 (− 0.587, 0.507)	0.89	− 0.14 (− 0.494, 0.213)	0.44
**MCP-1/Cr**	0.083 (− 0.538, 0.704)	0.79	0.289 (0.084, 0.494)	**0.006**	0.275 (− 0.301, 0.851)	0.35	–	–	–	–
**Clusterin**	−0.43 (− 0.981, 0.122)	0.13	0.106 (− 0.342, 0.555)	0.64	− 0.503 (−1.031, 0.024)	0.062	−0.273 (− 0.804, 0.258)	0.32	− 0.139 (− 0.566, 0.288)	0.52
**Clusterin/Cr**	−0.139 (− 0.654, 0.377)	0.60	−0.168 (− 0.56, 0.223)	0.40	−0.161 (− 0.651, 0.328)	0.52	–	–	–	–
**NGAL**	−0.025 (− 0.612, 0.562)	0.93	0.024 (− 0.171, 0.219)	0.81	−0.139 (− 0.673, 0.395)	0.61	−0.332 (− 0.671, 0.007)	0.055	0.16 (− 0.124, 0.443)	0.27
**NGAL/Cr**	0.172 (−0.386, 0.729)	0.55	−0.154 (− 0.35, 0.041)	0.12	0.117 (− 0.407, 0.641)	0.66	–	–	–	–
**8 IP**	−0.212 (− 0.439, 0.014)	0.067	0.208 (0.109, 0.308)	**<.001**	−0.242 (− 0.451, − 0.033)	**0.023**	0.082 (− 0.087, 0.251)	0.34	−0.141 (− 0.292, 0.01)	0.068
**8 IP/Cr**	0.045 (−0.098, 0.188)	0.54	0.043 (−0.021, 0.107)	0.19	0.107 (−0.034, 0.249)	0.14	–	–	–	–
**L-FABP**	0.011 (−0.211, 0.234)	0.92	0.053 (−0.026, 0.132)	0.19	0.099 (−0.118, 0.316)	0.37	−0.038 (− 0.163, 0.087)	0.55	− 0.018 (− 0.137, 0.1)	0.76
**L-FABP/Cr**	0.279 (− 0.01, 0.568)	0.059	− 0.165 (− 0.273, − 0.058)	**0.003**	0.421 (0.187, 0.654)	**<.001**	–	–	–	–
**OPN**	0.253 (− 0.383, 0.889)	0.44	0.134 (− 0.105, 0.373)	0.27	− 0.019 (− 0.629, 0.591)	0.95	0.03 (− 0.267, 0.327)	0.84	0.22 (− 0.319, 0.759)	0.42
**OPN/Cr**	0.514 (− 0.113, 1.14)	0.11	− 0.029 (− 0.261, 0.202)	0.81	0.345 (− 0.275, 0.966)	0.28	–	–	–	–
**H-FABP**	0.062 (− 0.23, 0.355)	0.68	0.149 (− 0.048, 0.346)	0.14	0.006 (− 0.305, 0.317)	0.97	− 0.067 (− 0.413, 0.279)	0.70	− 0.116 (− 0.335, 0.103)	0.30
**H-FABP/Cr**	0.32 (0.033, 0.607)	**0.029**	− 0.024 (− 0.214, 0.166)	0.81	0.353 (0.091, 0.615)	**0.008**	–	–	–	–

*P*-values in bold denote significance at the 0.05 level

Models were fit using the GEE procedure with the normal distribution, identity link and exchangeable correlation specifications, unless noted otherwise

Models were adjusted for age, sex and eGFR

^**a**^Only the urine concentrations unadjusted for creatinine were used since these variables are also adjusted for creatinine

^**b**^Log transformations were taken for all urine biomarkers as well as Uox, PTOx, Calcium/Cr and Citrate/Cr

Units of measure are Clusterin: ng/ml and μg/g Cr; NGAL: ng/ml and ng/g Cr; 8 IP: pg/ml and ng/g Cr; MCP1: pg/ml and pg/g Cr; L-FABP: ng/ml and μg/g Cr; OPN: ng/ml and μg/g Cr; H-FABP: pg/ml and ng/g Cr

**Table 4:** Association of urine markers with eGFR among *n* = 30 PH patients

Biomarker^**a**^	eGFR	
	Estimate (95% CI)	P
**Clusterin**	1.186 (− 0.42, 2.793)	0.15
**Clusterin/Cr**	1.377 (−0.347, 3.102)	0.12
**NGAL**	2.598 (1.118, 4.077)	**< 0.001**
**NGAL/Cr**	2.207 (0.56, 3.855)	**0.009**
**8 IP**	4.586 (0.05, 9.121)	0.054
**8 IP/Cr**	2.509 (−2.34, 7.358)	0.31
**MCP-1**	1.698 (−0.569, 3.966)	0.14
**MCP-1/Cr**	1.507 (−0.523, 3.536)	0.15
**L-FABP**	−3.542 (−10.156, 3.072)	0.29
**L-FABP/Cr**	−4.23 (−8.317, −0.143)	**0.043**
**OPN**	2.543 (0.751, 4.334)	**0.005**
**OPN/Cr**	2.223 (0.77, 3.676)	**0.003**
**H-FABP**	1.799 (−3.621, 7.218)	0.52
**H-FABP/Cr**	0.52 (−3.706, 4.746)	0.81

*P*-values in bold denote significance at the 0.05 level

Models were fit using the GEE procedure with the normal distribution, identity link and exchangeable correlation specifications, unless noted otherwise

Models were adjusted for age and sex

^a^Log transformations were taken for all urine biomarkers as well as Uox, PTOx, Calcium/Cr and Citrate/Cr

Units of measure are Clusterin: ng/ml and μg/g Cr; *NGAL* ng/ml and ng/g Cr, *8 IP* pg/ml and ng/g Cr, *MCP1* pg/ml and pg/g Cr, *L-FABP* ng/ml and μg/g Cr, *OPN* ng/ml and μg/g Cr, *H-FABP* pg/ml and ng/g Cr

An additional sensitivity analysis was conducted after excluding those serum and/or urine clinical laboratory values that were obtained more than 1 week away from the biobanked urine used for the biomarker testing (*n* = 7 serum values and *n* = 8 urine values). The estimates and significance remained essentially unchanged (Supplementary Tables 1 and 2).

## Discussion

In the current study, we used biobanked urine samples from a cohort of PH patients to determine the relationships between urinary biomarkers and renal function and urinary determinants of SS in this patient population. Urinary MCP-1 and 8 IP were both positively correlated with CaOx SS. Urinary excretions of other biomarkers including OPN and NGAL did not appear to associate with urinary excretions of oxalate or CaOx SS. Interestingly, however, urinary OPN and NGAL both positively associated with eGFR, suggesting their excretion might in part be related to intact renal mass.

MCP-1 is secreted following various stimuli by mononuclear cells and almost all types of intrinsic renal cells including mesangial cells, endothelial cells and tubular epithelial cells [17]. As an inflammatory chemokine, MCP-1 plays an important role in the development of kidney injury via recruitment and activation of monocytes/macrophages [17]. Studies have demonstrated that measurement of urinary MCP-1 has potential as a biomarker for diagnosis, prognosis and response to therapy in a variety of renal diseases including diabetic nephropathy, lupus nephritis, ANCA- associated vasculitis, and autosomal dominant polycystic kidney disease (ADPKD) [18–22]. Our recent study demonstrated that urine MCP-1 may even detect early tubulointerstitial fibrosis in living kidney donors with normal kidney function [22].

Earlier studies have demonstrated that exposure of cultured renal epithelial cells (NRK52E cells, a rat renal proximal tubular cell line) to calcium oxalate crystals resulted in increased expression of MCP-1 messenger RNA (mRNA) and protein [23]. Similar results were reported in HK-2 cells, an immortalized proximal tubule epithelial cell line from normal adult human kidney [24]. In addition, exposure of renal fibroblasts to oxalate ion and CaOx crystals increased MCP-1 mRNA and protein expression [25]. In vivo studies in hyperoxaluric model by using oxalate-fed wild type mice (not genetically modified PH mice model) have also demonstrated increased MCP-1 expression with increasing CaOx crystal deposition [26]. The present study provides evidence that similar pathways are activated in hyperoxaluric PH patients, and that urinary MCP-1 excretion is associated with CaOx SS. It’s unclear why urinary MCP-1 (and clusterin and OPN and H-FABP) excretions were overall greater in the control population than the PH cohort (Table 2). This might reflect overall difference in kidney function or mass between the two groups related to disease status or possibly age or other comorbidities.

Urinary 8IP was also positively associated with urinary CaOx SS in the PH cohort. Studies suggest that this marker of oxidative stress is released by cells in response to oxalate and CaOx crystals, and is associated with superoxide and increased expression of NAPDH oxidase activity [27]. Previous studies demonstrated that lipid peroxides are increased in the kidneys and urine of hyperoxaluric rats fed ethylene glycol [28], and these values normalized and renal calcium oxalate crystal deposition improved with the co-administration of the antioxidant vitamin E [29]. Use of the NAPDH oxidase inhibitor apocyanin also reduced oxidative stress and crystal deposition in hyperoxaluric rat models [30]. The resulting oxidative stress and inflammation in these rodents is associated with increased MCP-1 gene and protein expression [31].

The urine of idiopathic calcium oxalate stone formers has increased amounts of N-acetyl-β-glucoseaminidase, α-glutathione S-transferase, malondialdehyde, and thiobarbituric acid reactive substances, suggesting a link between oxidative stress and stone disease in humans that parallels the observations in the hyperoxaluric rodent models [32]. Interestingly, in our study of PH patients, who are as a rule are the most hyperoxaluric of CaOx stone formers, urinary 8 IP was negatively associated with PTOx and urinary oxalate excretion, but positively correlated with CaOx SS (Table 3). These findings might suggest that CaOx crystals (as opposed to oxalate ion) are the key stimulus for oxidative stress in these hyperoxaluric conditions. Indeed, since cell culture medium is high in calcium, exogenous addition of oxalate invariably leads to calcium oxalate crystallization, and studies to date have not effectively teased apart the effects of oxalate ion versus CaOx crystals.

Our data also suggest that higher levels of urine OPN and NGAL are associated with better kidney function. The underlying mechanism(s) may deserve further studies. OPN is a phosphorylated protein of wide tissue distribution that is found in association with dystrophic calcification including in the organic matrix of kidney stones. Although there is still debate regarding its effect upon crystal adhesion to tubular epithelial cells, studies have clearly demonstrated that OPN is a strong inhibitor of crystal formation and growth in vitro [33]. In the current study, urine levels of OPN were not independently associated with oxalate excretion, PTOx, or CaOx crystallization. Although the relatively small sample size could be a factor, it is also possible that urinary excretion of these biomarkers is also influenced by the amount of healthy renal mass, as reflected by eGFR. A similar rationale might be applied to urinary NGAL in the current study, which appears to have potential as a biomarker for acute kidney injury [34] and as a highly sensitive and specific predictor of systemic inflammatory response syndrome for patients presenting with nephrolithiasis [35]. In the case of OPN, it is also possible that CaOx crystallization could upregulate its production, but it is then incorporated into the developing crystals rather that excreted into the urine. Future prospective studies with larger group sizes may help further define the roles of osteopontin and NGAL in ongoing renal damage in PH.

Our study has certain limitations. The number of patient samples was limited and they were not collected on a protocolized basis; thus results may have been biased towards those with more severe disease. However, this is one of the larger cohorts of patients with the rare disorder of PH to have a comprehensive panel of kidney injury biomarkers assessed. Furthermore, we lacked detailed clinical data from the control population including recent dietary intakes. However, the subjects lacked a clinical diagnosis of kidney disease or kidney stone disease and were a similar subset of the group used to define the reference ranges in our laboratory for kidney stone analytes. Thus, we have no reason to suspect that there urine composition would different any systematic way from the reference values in our laboratory.

## Conclusions

Although the mechanisms are not entirely clear, CaOx crystallization in renal tubules has been considered as one of the critical therapeutic targets in the development of new therapeutic approach to treat PH. The association between urine level of MCP-1 and CaOx crystallization may suggest urine MCP-1 could be used as a marker to assist the evaluation for the effectiveness of treatments in PH patients. In addition, this association may suggest that MCP-1 signaling pathway might be in involved in the etiology of renal damage in PH. Further prospective studies are warranted to determine whether urine excretion of MCP-1 can predict long term outcomes.

## Supplementary information

**Additional file 1 : Supplemental Table 1** Association of urine markers with 24 urine oxalate excretion (Uox), CaOx supersaturation (CaOx SS), proximal tubular oxalate concentration (PTOx), urine calcium/Cr, and urine citrate/Cr, among *n* = 30 PH patients, after removing those with urine laboratory values obtained more than 1 week away from the corresponding urine biobank specimen. *P*-values in bold denote significance at the 0.05 level. Models were fit using the GEE procedure with the normal distribution, identity link and exchangeable correlation specifications, unless noted otherwise. Models were adjusted for age, sex and eGFR. @ Only the urine concentrations unadjusted for creatinine were used since these variables are also adjusted for creatinine. **‡**Log transformations were taken for all urine biomarkers as well as Uox, PTOx, Calcium/Cr and Citrate/Cr. Units of measure are Clusterin: ng/ml and μg/g Cr; NGAL: ng/ml and ng/g Cr; 8 IP: pg/ml and ng/g Cr; MCP1: pg/ml and pg/g Cr; L-FABP: ng/ml and μg/g Cr; OPN: ng/ml and μg/g Cr; H-FABP: pg/ml and ng/g Cr.

**Additional file 2 **: **Supplemental Table 2**. Association of urine markers with eGFR among *n* = 30 PH patients, after removing those with serum creatinine values obtained more than 1 week away from the corresponding urine biobank specimen. *P*-values in bold denote significance at the 0.05 level. Models were fit using the GEE procedure with the normal distribution, identity link and exchangeable correlation specifications, unless noted otherwise. Models were adjusted for age and sex. ‡Log transformations were taken for all urine biomarkers as well as Uox, PTOx, Calcium/Cr and Citrate/Cr Units of measure are Clusterin: ng/ml and μg/g Cr; NGAL: ng/ml and ng/g Cr; 8 IP: pg/ml and ng/g Cr; MCP1: pg/ml and pg/g Cr; L-FABP: ng/ml and μg/g Cr; OPN: ng/ml and μg/g Cr; H-FABP: pg/ml and ng/g Cr.

## Data Availability

The datasets used and/or analyzed during the current study are available from the corresponding author on reasonable request.

## Abbreviations

AGT: Alanine-glyoxylate aminotransferase protein
*AGXT*: Alanine-glyoxylate aminotransferase gene
BSA: Body surface area
CaOx SS: Calcium oxalate supersaturation
CCL2: Chemokine (C-C motif) ligand 2
CI: Confidence interval
CKD: Chronic kidney disease
Cr: Creatinine
D.G.: Delta Gibbs free energy unit
eGFR: Estimated glomerular filtration rate
ESRD: End stage renal disease
GEE: Generalized estimating equation
GRHPR: Glyoxylate and hydroxypyruvate reductase
H-FABP: Liver-type fatty acid binding protein
HOGA1: 4-hydroxy-2-oxoglutarate aldolase
L-FABP: Liver-type fatty acid binding protein
8IP: 8-isoprostane
MCP-1: Monocyte chemoattractant protein-1
mRNA: Messenger RNA
NGAL: Neutrophil gelatinase-associated lipocalin
OR: Odds ratio
OPN: Osteopontin
PH: Primary hyperoxaluria
PTOx: Calculated proximal tubular oxalate concentration
RKSC: Rare kidney stone consortium
RNAi: Ribonucleic acid inhibition
UOx: Urinary oxalate
