# Supplementary material for: Urinary monocyte chemoattractant protein 1 associated with calcium oxalate crystallization in patients with primary hyperoxaluria
Source: BMC Nephrol. 2020 Apr 15;21:133. doi: 10.1186/s12882-020-01783-z (PMC7161151; doi:10.1186/s12882-020-01783-z)
Supplement: Supplementary file 1 — Additional file 1 : Supplemental Table 1 Association of urine markers with 24 urine oxalate excretion (Uox), CaOx supersaturation (CaOx SS), proximal tubular oxalate concentration (PTOx), urine calcium/Cr, and urine citrate/Cr, among n = 30 PH patients, after removing those with urine laboratory values obtained more than 1 week away from the corresponding urine biobank specimen. P-values in bold denote significance at the 0.05 level. Models were fit using the GEE procedure with the normal distribution, identity link and exchangeable correlation specifications, unless noted otherwise. Models were adjusted for age, sex and eGFR. @ Only the urine concentrations unadjusted for creatinine were used since these variables are also adjusted for creatinine. ‡Log transformations were taken for all urine biomarkers as well as Uox, PTOx, Calcium/Cr and Citrate/Cr. Units of measure are Clusterin: ng/ml and μg/g Cr; NGAL: ng/ml and ng/g Cr; 8 IP: pg/ml and ng/g Cr; MCP1: pg/ml and pg/g Cr; L-FABP: ng/ml and μg/g Cr; OPN: ng/ml and μg/g Cr; H-FABP: pg/ml and ng/g Cr. [file 12882_2020_1783_MOESM1_ESM.docx]

**Supplemental Table 1. Association of urine markers with 24 urine oxalate excretion (Uox), CaOx supersaturation( CaOx SS), proximal tubular oxalate concentration( PTOx), urine calcium/Cr, and urine citrate/Cr, among n=30 PH patients, after removing those with urine laboratory values obtained more than one week away from the corresponding urine biobank specimen.**

|  | **Uox‡** | | **CaOx SS** | | **PTOx@‡** | | **Calcium/ Cr@‡** | | **Citrate/ Cr@‡** | |
| --- | --- | --- | --- | --- | --- | --- | --- | --- | --- | --- |
| **Biomarker‡** | **Estimate (95% CI)** | **P** | **Estimate (95% CI)** | **P** | **Estimate (95% CI)** | **P** | **Estimate (95% CI)** | **P** | **Estimate (95% CI)** | **P** |
| **MCP-1** | -0.093 (-0.842, 0.657) | 0.81 | 0.485 (0.213, 0.756) | **<0.001** | -0.1 (-0.764, 0.564) | 0.77 | -0.095 (-0.627, 0.438) | 0.73 | -0.217 (-0.549, 0.115) | 0.20 |
| **MCP-1/Cr** | 0.239 (-0.406, 0.883) | 0.47 | 0.314 (0.079, 0.549) | **0.009** | 0.371 (-0.236, 0.979) | 0.23 |  |  |  |  |
| **Clusterin** | -0.594 (-1.181, -0.006) | **0.048** | 0.09 (-0.45, 0.631) | 0.74 | -0.567 (-1.132, -0.002) | **0.049** | -0.318 (-0.864, 0.228) | 0.25 | -0.198 (-0.658, 0.263) | 0.40 |
| **Clusterin/Cr** | -0.262 (-0.801, 0.276) | 0.34 | -0.228 (-0.662, 0.207) | 0.30 | -0.178 (-0.699, 0.344) | 0.51 |  |  |  |  |
| **NGAL** | 0.009 (-0.586, 0.604) | 0.98 | -0.017 (-0.228, 0.194) | 0.88 | -0.114 (-0.632, 0.404) | 0.67 | -0.4 (-0.734, -0.067) | **0.019** | 0.116 (-0.186, 0.418) | 0.45 |
| **NGAL/Cr** | 0.252 (-0.325, 0.828) | 0.39 | -0.204 (-0.423, 0.015) | 0.07 | 0.221 (-0.285, 0.726) | 0.39 |  |  |  |  |
| **8 IP** | -0.231 (-0.466, 0.003) | 0.053 | 0.245 (0.121, 0.368) | **<0.001** | -0.272 (-0.48, -0.063) | **0.012** | 0.09 (-0.092, 0.271) | 0.33 | -0.146 (-0.302, 0.01) | 0.066 |
| **8 IP/Cr** | 0.062 (-0.085, 0.209) | 0.41 | 0.061 (-0.019, 0.14) | 0.13 | 0.115 (-0.025, 0.256) | 0.11 |  |  |  |  |
| **L-FABP** | 0.032 (-0.192, 0.255) | 0.78 | 0.044 (-0.037, 0.125) | 0.29 | 0.125 (-0.092, 0.341) | 0.26 | -0.059 (-0.188, 0.071) | 0.37 | -0.002 (-0.128, 0.124) | 0.98 |
| **L-FABP/Cr** | 0.347 (0.091, 0.603) | **0.008** | -0.183 (-0.311, -0.055) | **0.005** | 0.501 (0.3, 0.701) | **<0.001** |  |  |  |  |
| **OPN** | 0.228 (-0.443, 0.899) | 0.51 | 0.18 (-0.072, 0.432) | 0.16 | -0.027 (-0.676, 0.622) | 0.94 | 0.065 (-0.251, 0.382) | 0.69 | 0.209 (-0.374, 0.791) | 0.48 |
| **OPN/Cr** | 0.524 (-0.142, 1.189) | 0.12 | 0.007 (-0.232, 0.246) | 0.96 | 0.407 (-0.246, 1.06) | 0.22 |  |  |  |  |
| **H-FABP** | -0.023 (-0.3, 0.253) | 0.87 | 0.109 (-0.098, 0.316) | 0.30 | -0.092 (-0.377, 0.192) | 0.53 | -0.11 (-0.449, 0.228) | 0.52 | -0.092 (-0.292, 0.107) | 0.36 |
| **H-FABP/Cr** | 0.277 (0.008, 0.547) | **0.044** | -0.068 (-0.271, 0.134) | 0.51 | 0.31 (0.055, 0.565) | **0.017** |  |  |  |  |

P-values in bold denote significance at the 0.05 level.

Models were fit using the GEE procedure with the normal distribution, identity link and exchangeable correlation specifications, unless noted otherwise.

Models were adjusted for age, sex and eGFR.

@ Only the urine concentrations unadjusted for creatinine were used since these variables are also adjusted for creatinine.

**‡**Log transformations were taken for all urine biomarkers as well as Uox, PTOx, Calcium/Cr and Citrate/Cr

Units of measure are Clusterin: ng/ml and µg/g Cr; NGAL: ng/ml and ng/g Cr; 8 IP: pg/ml and ng/g Cr; MCP1: pg/ml and pg/g Cr; L-FABP: ng/ml and µg/g Cr; OPN: ng/ml and µg/g Cr; H-FABP: pg/ml and ng/g Cr.
